# Vomerine hamartoma in a cleft palate child

**DOI:** 10.4103/0970-0358.73462

**Published:** 2010

**Authors:** Rameshwar L. Bang, Hisham Burezq, Imad Al-Najjadah

**Affiliations:** 1 Al Babtain Centre for Burns and Plastic Surgery, Ibn-Sina Hospital Kuwait; 2 Department of Surgery, Faculty of Medicine, Kuwait University, Kuwait

**Keywords:** Cleft palate, hamartoma, vomer

## Abstract

A case of a female child born at full term after normal vaginal delivery with bilateral secondary complete cleft palate and vomerine hamartoma mimicking intra-oral midline encephalocoele. Radiologically the tumour was confined to the vomer without intra-cranial extension. The lesion was occupying the oral cavity causing feeding problem. Surgical excision of the tumour at the age of six months and two flaps palatoplasty at the age of twelve months were performed. On histopathology the lesion turned out to be a lipomatous hamartoma of a benign nature. The child was followed for 9 years with no evidence of recurrence and a satisfactory speech. To our knowledge this is the first report of cleft palate with vomerine hamartoma in the English literature.

## INTRODUCTION

Congenital malformations may be the product of errors in embryogenesis or the result of intrauterine events disturbing embryonic and foetal growth. Hamartomas and teratomas presenting at birth are tumour-like malformations of anatomical developmental error.[1–4] Hamartomas in the form of melanocytic nevi or vascular malformations such as haemangiomas and lymphatic malformations are quite common in the head and neck but lesions composed of other tissue types are rare.[1,2,4–6] Hamartomas located on the tongue near the foramen cecum or on the hard palate near the incisive papilla are the usual occurrence,[1–10] but one arising from the vomer and associated with cleft palate was not reported earlier. Large or rapidly growing hamartomas of either the palate or the tongue can cause difficulty in feeding or breathing in an infant. Generally these lesions are benign in nature and simple excision is curative. A case of a vomerine hamartoma and bilateral cleft palate is being presented.

## CASE REPORT

A female child, a product of a full term spontaneous normal vaginal delivery, with a birth weight of 3.500 kg and the Apgar at birth, 5, and 15 minutes was 9, 11, and 14, respectively had a positive parental consanguinity and positive family history of cleft palate. She presented to our clinic at the age of four days. Clinical examination revealed bilateral complete cleft palate and a midline palatal mass extending to the palatal shelves laterally and alveolar arch anteriorly measuring about 4cm × 3cm × 0.5cm [Figure 1]. The lip and the alveolus were normal. The diagnosis of meningo-encephalocoele was considered though the expansile impulse on crying was absent. X-ray skull was within the normal range. Oral feeding was difficult and so we opted for nasogastric feding. Although the mass was gradually increasing in size, there were no airway problems and the child continued to thrive well. CT scan at the age of six weeks showed no skull base bone defect and no evidence of meningo-encephalocoele.

**Figure 1a:**
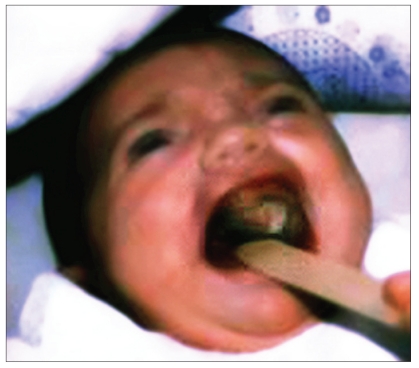
Preoperative intra-oral view showing a midline palatal lesion extending more to the left side.

**Figure 1b:**
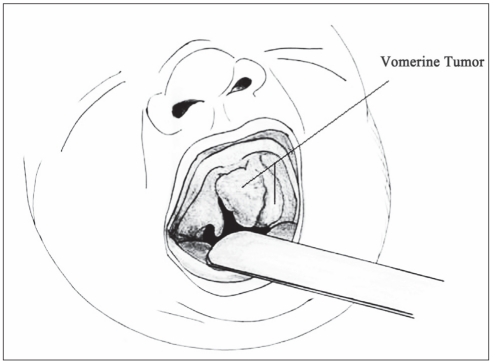
Sketch of the same photo.

**Figure 2:**
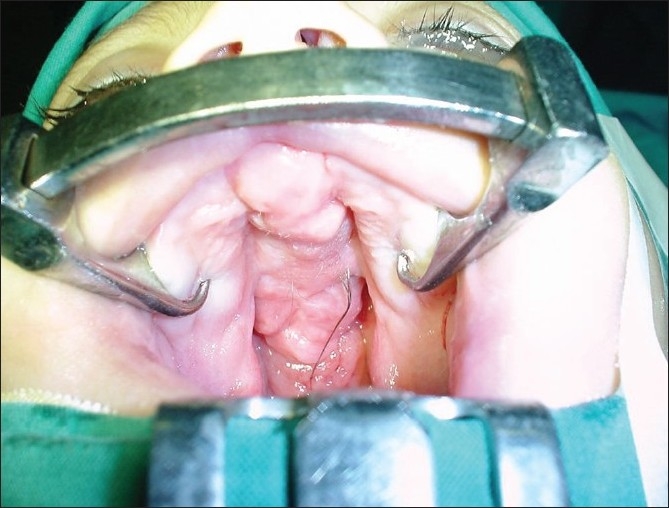
Post-operative intra-oral view after tumour excision.

**Figure 3:**
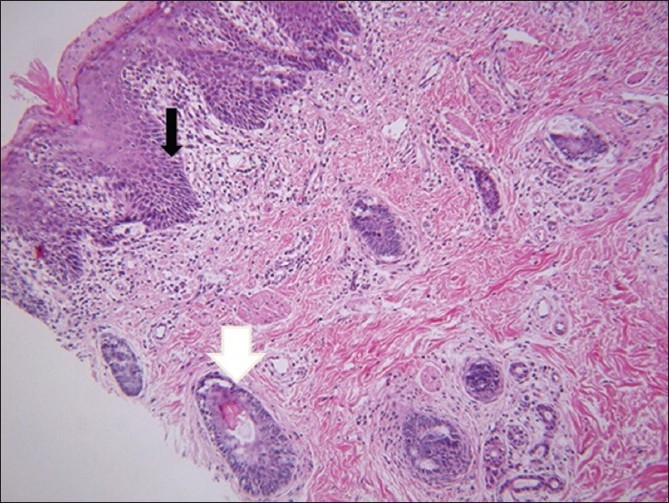
Photomicrograph of the excised lesion shows a covering of mature stratified squamous epitheliums identical to skin epithelium (black arrow). Hair follicles (white arrow) and collections of sweat glands (small black arrow) are also present. (Paraffin sections 5 microns H & E ×100).

**Figure 4:**
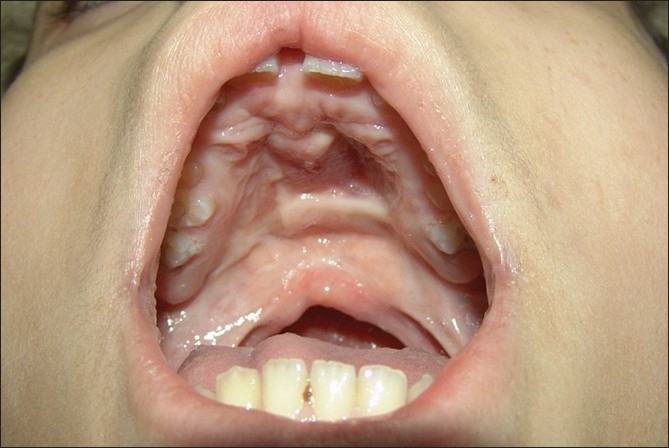
Palate shown at the age of 10 years.

At the age of 6 months, under general anaesthesia and endotracheal intubation the tumour mass was excised leaving a sleeve of mucosal tissues on either side for closure [Figure 2]. Histopathology showed skin and buccal mucosa with both vellus and terminal hair shafts, immature hair follicles, and rare poorly developed sebaceous glands. There was a copious amount of mature adipose tissue below the skin and buccal mucosa. A variable amount of fibrous septae and vessels were present in the adipose tissue suggestive of lipomatous hamartoma [Figure 3]. The child recovered well and normal oral feeding was possible 3 weeks after surgery. At the age of 12 month the closure of palate was done by two flaps palatoplasty; this was complicated by anterior palatal fistula which was repaired around the age of three years. The child was followed for 9 years and showed no evidence of recurrence of the tumour [Figure 4].and had an acceptable speech

## DISCUSSION

Hamartoma of the oral cavity is a rare tumour.[1–10] The lesion observed in this patient certainly raised the suspicion of a midline encephalocoele and a differential diagnosis of teratoma, and hamartoma was considered. Midline encephalocoele is far more serious condition as compared to benign lipomatous hamartomas. Napier *et al*.[1] reported a case of unusual leiomyomatous hamartoma of the hard palate in a normal five-year-old girl and saw no recurrence following surgical excision. Harada *et al*.,[6] Piattelli *et al*.,[9] De Biase *et al*.,[2] Liang *et al*.,[10] Takeyama *et al*.,[3] and Zaitoum *et al*.[4] made the similar observation of a hamartomatous lesion on a hard palate. In our patient the congenital lesion was associated with cleft palate but with no evidence of abnormal salivary gland tissues.

Takeyama *et al*.[3] described a case of hamartoma on the hard palate associated with corpus callosum agenesis, microphthalmia, and skin malformation. Goldsmith *et al*.[8] has reported a case of leiomyomatous hamartoma of the posterior tongue in a 16-month-old child causing dysphagia and this was resolved after excision of the tumour as observed in the described case. Miyamoto *et al*.[1] reported a lingual hamartoma in a child with a cleft palate. Lingual hamartoma is much more common compared to hard palate lesions. The hamartoma in the oral cavity is usually located on the tongue near the foramen cecum or the incisive papilla of the hard palate, which might be related to the line of fusion.

Having a cleft palate and a vomerine or palatal tumour would be preferably managed by starting with tumour excision at the age of 3–6 months. This would solve the problem of dysphagia or dyspnoea if present, give more time to study the lesion histologically, and to evaluate the risk of recurrence or the need for re-excision. Consequently, definitive treatment of the cleft can take place later at the age of 9–12 months. More reports of these rare tumours are needed to understand their nature and behaviour.
